# Comparative Outcomes of Heart Failure with Preserved vs. Mildly Reduced Ejection Fraction After Off-Pump Coronary Artery Bypass: A Single-Center Retrospective Study

**DOI:** 10.3390/jcm15186946

**Published:** 2026-09-08

**Authors:** Do Jung Kim, Seungji Hyun, Hyelynn Jeon, Bumhee Park, You Sun Hong, Sang Hyun Lim

**Affiliations:** 1 Department of Thoracic and Cardiovascular Surgery, Ajou University Medical Center, Ajou University School of Medicine, 164 World cup-ro, Yeongtong-gu, Suwon 16499, Republic of Koreagustmdwl0723@naver.com (S.H.); yshongjin@aumc.ac.kr (Y.S.H.); 2 Office of Biostatistics, Medical Research Collaborating Center, Ajou Research Institute for Innovative Medicine, Ajou University Medical Center, 164 World cup-ro, Yeongtong-gu, Suwon 16499, Republic of Korea; wjsur1028@gmail.com (H.J.); bhpark@ajou.ac.kr (B.P.)

**Keywords:** coronary artery bypass grafting, heart failure, mildly reduced ejection fraction, preserved ejection fraction, clinical outcomes

## Abstract

**Background/Objectives:** Heart failure (HF) phenotype based on left ventricular ejection fraction (LVEF) may be associated with outcomes after coronary revascularization, but data comparing patients with HF with preserved EF (HFpEF) and HF with mildly reduced EF (HFmrEF) undergoing coronary artery bypass grafting (CABG) remain limited. We compared clinical outcomes and longitudinal echocardiographic changes between patients with HFpEF and HFmrEF undergoing isolated off-pump CABG (OPCAB). **Methods:** We retrospectively analyzed 362 patients with HF who underwent isolated OPCAB between 2012 and 2020, including 273 with HFpEF (LVEF ≥ 50%) and 89 with HFmrEF (LVEF 41–49%). Overall survival was analyzed using Cox regression, and composite cardiovascular adverse events (CCAEs) and their individual components were analyzed using Fine–Gray models. Inverse probability of treatment weighting (IPTW) was used to reduce measured baseline confounding. Longitudinal echocardiographic changes were evaluated using linear mixed-effects models. **Results:** Patients with HFmrEF had higher baseline NT-proBNP levels and more adverse echocardiographic features than those with HFpEF. In IPTW-weighted analyses, HFmrEF was associated with all-cause mortality (HR, 3.09; 95% CI, 1.39–6.89; *p* = 0.006), HF readmission (sHR, 5.72; 95% CI, 2.12–15.45; *p* < 0.001), and CCAEs (sHR, 2.67; 95% CI, 1.37–5.19; *p* = 0.004). A significant group-by-time interaction was observed for LVEF (*p* < 0.001), with an early postoperative increase in LVEF in the HFmrEF group. **Conclusions:** In patients with HF undergoing isolated OPCAB at a single center, HFmrEF was associated with poorer long-term outcomes than HFpEF, although an early postoperative increase in LVEF was observed. These associations may partly reflect differences in baseline HF severity rather than EF category alone.

## 1. Introduction

Heart failure (HF) is a common and clinically important manifestation of ischemic heart disease, particularly in patients with advanced coronary artery disease (CAD) requiring revascularization [1,2]. Coronary artery bypass grafting (CABG) is an established revascularization strategy for selected patients with HF and left main or multivessel CAD [3]. Although HF with reduced ejection fraction (EF; HFrEF) is recognized for its unfavorable short- and long-term prognoses, the Surgical Treatment for Ischemic Heart Failure Extension Study demonstrated a significant 10-year survival benefit when CABG was added to optimal medical therapy in patients with severe left ventricular (LV) systolic dysfunction (LVEF < 35%) [4]. According to contemporary HF guidelines [5], patients with EFs between 41% and 49% are classified as having HF with mildly reduced EF (HFmrEF), accounting for approximately 25% of all HF cases [5,6,7]. Recent studies suggest that patients with HFmrEF share clinical characteristics and treatment responses more closely with those of HFrEF than with those of HF with preserved EF (HFpEF), particularly in the context of ischemic heart disease [6,8]. Previous studies have demonstrated progressively poorer outcomes with decreasing LVEF after CABG [9,10,11]. However, LVEF has generally been evaluated as a continuous measure or using broader EF categories, and direct comparisons between HFmrEF and HFpEF among patients with clinically identified HF undergoing CABG remain limited.

Therefore, this study aimed to compare long-term clinical outcomes, including all-cause mortality and composite cardiovascular adverse events (CCAEs), and longitudinal echocardiographic changes between patients with HFmrEF and HFpEF undergoing isolated off-pump CABG (OPCAB).

## 2. Materials and Methods

### 2.1. Study Population

**Figure 1:**
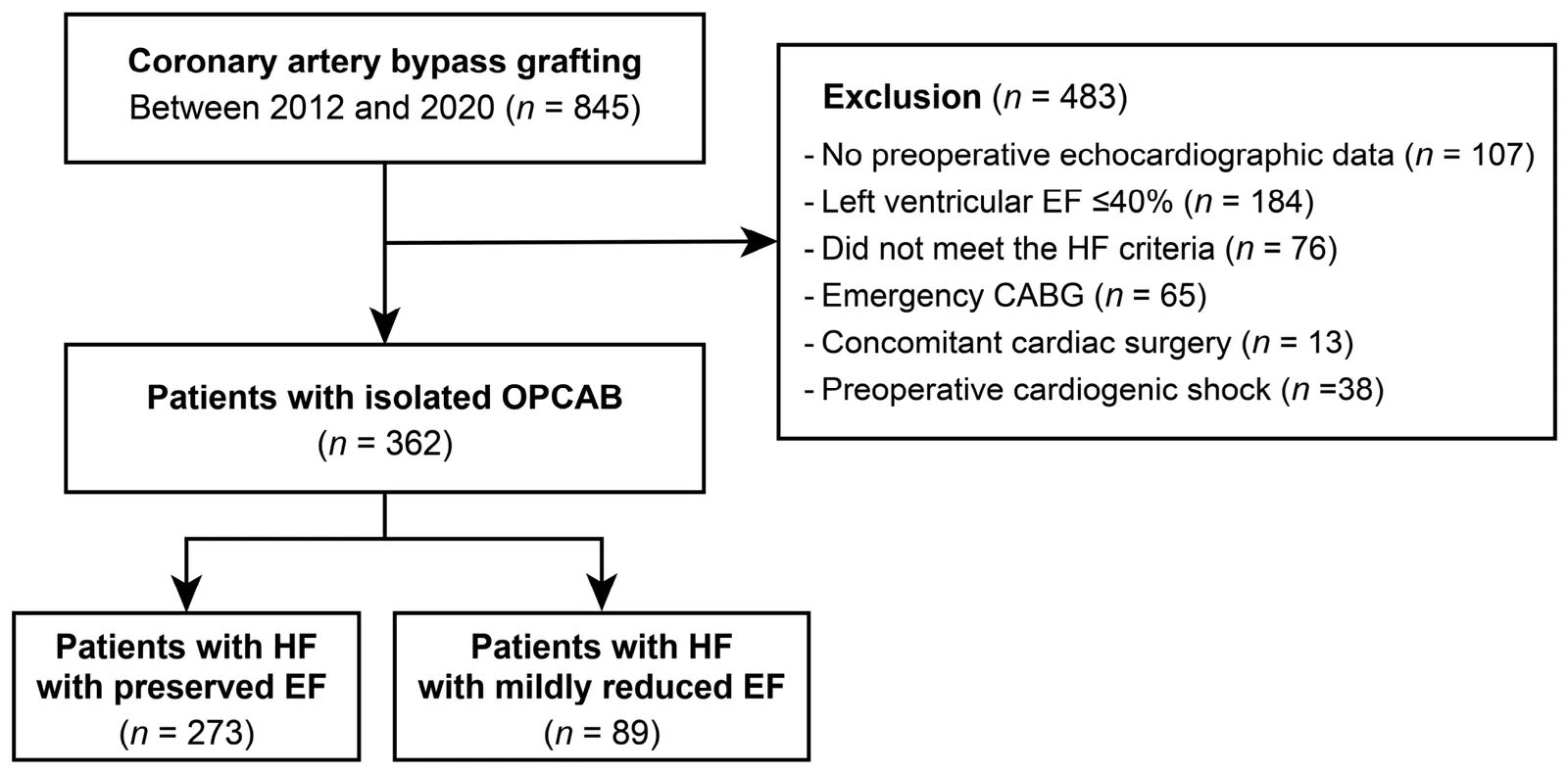
Flow diagram of patient selection. Exclusion criteria were applied sequentially, with patients meeting multiple criteria counted only at the first applicable step. Among the initial cohort, 54 patients underwent on-pump CABG (35 planned and 19 converted from OPCAB), all of whom were excluded through these criteria. CABG, coronary artery bypass grafting; EF, ejection fraction; HF, heart failure; OPCAB, off-pump coronary artery bypass grafting.

From January 2012 to December 2020, we retrospectively identified 845 consecutive patients who underwent CABG at Ajou University Hospital, Ajou University School of Medicine. Patients were sequentially excluded if they lacked preoperative echocardiographic data (*n* = 107), had an LVEF ≤ 40% (*n* = 184), did not meet the criteria for HF (*n* = 76), underwent emergency CABG (*n* = 65) or concomitant cardiac surgery (*n* = 13), or had preoperative cardiogenic shock (*n* = 38) (Figure 1). Patients meeting more than one exclusion criterion were counted only at the first applicable step. Among the initial cohort, 54 patients underwent on-pump CABG (35 planned and 19 converted from OPCAB); all met at least one of the sequential exclusion criteria and were therefore excluded from the final OPCAB cohort.

HF was retrospectively ascertained from preoperative medical records based on documented symptoms and/or signs consistent with HF, including exertional dyspnea, orthopnea, peripheral edema, or reduced exercise tolerance, together with at least one of the following available preoperative findings: E/e′ > 9, increased LV mass index (≥115 g/m^2^ in men or ≥95 g/m^2^ in women), tricuspid regurgitation velocity > 2.8 m/s, or NT-proBNP > 125 pg/mL [5]. For patients with available NT-proBNP measurements, the value obtained closest to the date of surgery during the preoperative period was used. All included patients met at least one of these objective criteria. NYHA functional class was not systematically available for all patients and was therefore not used as a diagnostic criterion. Based on preoperative LVEF, the final cohort comprised 273 patients with HFpEF (≥50%) and 89 with HFmrEF (41–49%).

This retrospective study was conducted in accordance with the Declaration of Helsinki and was approved by the Institutional Review Board of Ajou University Hospital (AJOUIRB-DB-2023-599). The requirement for individual informed consent was waived by the Institutional Review Board because the study involved retrospective analysis of existing clinical data.

### 2.2. Surgical Technique

OPCAB was the routine approach for isolated CABG at our institution during the study period, whereas cardiopulmonary bypass was used selectively according to ventricular function, hemodynamic status, and intraoperative circumstances. Selection of target vessels and conduits was individualized by the operating surgeon, taking into account coronary vessel size and stenosis severity, the condition of the ascending aorta, and patient comorbidities. Complete revascularization was defined as bypass grafting of all significantly stenosed coronary vessels considered suitable for grafting. Through a standard median sternotomy, one or both internal thoracic arteries (ITAs) were harvested with a semi-skeletonized technique. Preoperative Doppler ultrasonography was used to assess potential additional conduits, including the radial artery and saphenous vein. The left ITA was preferentially used as an in situ graft to the left anterior descending artery. When a proximal aortic anastomosis was required, a Heartstring proximal seal device (Getinge, Wayne, NJ, USA) was used on a non-calcified portion of the ascending aorta, with the anastomosis constructed using continuous 6-0 polypropylene suture. Composite Y grafts were constructed between the left ITA and an additional conduit using continuous 8-0 polypropylene suture. Additional conduits were directed to appropriate targets within the left anterior descending, left circumflex, or right coronary artery territories.

After OPCAB, antiplatelet agents, beta-blockers, and statins were routinely prescribed unless contraindicated. Angiotensin receptor blockers or angiotensin-converting enzyme inhibitors were additionally prescribed when clinically indicated.

### 2.3. Echocardiographic Assessment

Echocardiographic measurements were obtained from routine clinical reports. Standard two-dimensional and Doppler echocardiography was performed according to the recommendations of the American Society of Echocardiography and the European Association of Cardiovascular Imaging [12,13]. LVEF and left atrial volume were assessed using the biplane modified Simpson’s method from apical four- and two-chamber views, with left atrial volume indexed to body surface area to obtain LAVI. LV mass was calculated using the Devereux-modified cube formula and indexed to body surface area to obtain LVMI. Transmitral E and septal e′ velocities were measured using pulsed-wave and tissue Doppler imaging, respectively, and E/e′ was calculated as their ratio.

### 2.4. Follow-Up and Study Endpoints

Baseline and perioperative variables were retrieved retrospectively from the institutional cardiac and vascular research databases. Survival status and date of death were ascertained through the Korea National Statistical Office database and were available for the entire cohort. Non-fatal clinical events, including HF readmission and coronary reintervention, were retrospectively identified from institutional medical records without independent adjudication. Follow-up data were censored on 31 December 2022. The median follow-up duration for survival was 4.7 (IQR, 2.97–6.64) years. Echocardiograms were evaluated preoperatively, before discharge, and preferably annually thereafter, with each follow-up year through Year 9 defined using a ±6-month window. The median echocardiographic follow-up duration was 4.5 (IQR, 2.83–7.15) years.

The clinical endpoints were all-cause death, cardiac death, HF readmission, coronary reintervention, and CCAEs. HF readmission was defined as an unplanned hospitalization for worsening HF symptoms and/or signs requiring initiation or intensification of HF-directed treatment. Coronary reintervention was defined as clinically driven repeat coronary revascularization (percutaneous coronary intervention or CABG) after the index CABG for recurrent symptoms and/or objective evidence of myocardial ischemia, excluding planned staged procedures. CCAEs were defined as the first occurrence of cardiac death, HF readmission, or coronary reintervention. Cardiac death was defined as death attributable to a cardiac cause, including sudden cardiac death.

### 2.5. Statistical Analysis

All analyses were performed with R 4.3.0 (R Core Development Team, Vienna, Austria) and SPSS Statistics 23.0 (IBM Corp., Armonk, NY, USA). Normality of continuous data was evaluated with the Shapiro–Wilk test. Depending on their distribution, continuous variables are presented as mean ± standard deviation or median (interquartile range), and between-group comparisons were made with Student’s *t*-test or the Mann–Whitney U test. Categorical data are presented as numbers (%) and were compared using the χ^2^ or Fisher’s exact test, as appropriate.

Overall survival was analyzed using Kaplan–Meier estimates and Cox proportional hazards models. Cumulative incidence functions and Fine–Gray models were used for competing-risk outcomes, with subdistribution hazard ratios (sHRs) and 95% confidence intervals (CIs) reported. All-cause death was the competing event for HF readmission and coronary reintervention, and non-cardiac death for cardiac death and CCAEs.

Given the limited number of outcome events, multivariable Cox and Fine–Gray models were parsimoniously adjusted for HF phenotype, age, chronic renal failure, and peripheral arterial disease. Multiple correlated echocardiographic measures were not simultaneously included given concerns regarding overfitting and multicollinearity, and NT-proBNP was excluded because it was available only in a subset of patients.

To reduce measured baseline confounding, inverse probability of treatment weighting (IPTW) was applied using propensity scores estimated by multivariable logistic regression with 13 baseline covariates: age, female sex, body mass index, smoking, hypertension, diabetes mellitus, chronic renal failure, cerebrovascular accident, peripheral arterial disease, dyslipidemia, previous percutaneous coronary intervention, three-vessel disease, and left main disease. Inverse probability weights were calculated to estimate the average treatment effect (ATE). Unstabilized weights were used, and no trimming or truncation was applied. Propensity-score distributions and IPTW weight distributions were examined to assess overlap and potentially influential weights, respectively. Covariate balance was evaluated using standardized mean differences (SMDs) before and after weighting, with an absolute SMD < 0.1 indicating adequate balance. IPTW-weighted Cox and Fine–Gray models were then used to estimate HRs and sHRs for HF phenotype, respectively, with robust standard errors clustered by patient ID.

The proportional hazards assumption for the Cox models was assessed using Schoenfeld residuals, and the proportional subdistribution hazards assumption for the Fine–Gray models was assessed using Schoenfeld residuals and covariate-by-time interaction tests. Longitudinal changes in echocardiographic parameters were evaluated using linear mixed-effects models including group, time, and group-by-time interaction terms, with time treated as a categorical variable. All available repeated measurements were included without imputation or interpolation of missing data. All tests were two-sided, and a *p* value < 0.05 was considered statistically significant.

## 3. Results

### 3.1. Baseline Characteristics

Baseline clinical characteristics and covariate balance before and after IPTW are presented in Table 1. Before weighting, peripheral arterial disease was more frequent in the HFmrEF group than in the HFpEF group (9.0% vs. 2.6%, *p* = 0.014). The propensity-score distributions showed adequate overlap between the HFpEF and HFmrEF groups (Supplementary Figure S1). After IPTW, all 13 covariates included in the propensity-score model achieved adequate balance between the groups, with absolute SMDs < 0.1 (Supplementary Figure S2).

Preoperative biomarker and echocardiographic characteristics are presented in Table 2. Among patients with available NT-proBNP measurements (*n* = 234), NT-proBNP levels were higher in the HFmrEF group than in the HFpEF group (1909 [745–8202] vs. 178 [136–619] pg/mL, *p* < 0.001). Patients with HFmrEF also had lower LVEF, larger LVESD and LVEDD, higher LVMI, and higher E/e′ (all *p* ≤ 0.016).

### 3.2. Operative and Clinical Outcomes

Operative characteristics and clinical outcomes are summarized in Table 3. Operative characteristics were generally similar between the groups. Complete revascularization was achieved in 93.8% of patients with HFpEF and 87.6% of those with HFmrEF (*p* = 0.069). Newly required dialysis was more frequent in the HFmrEF group (7.9% vs. 1.8%, *p* = 0.012), whereas in-hospital mortality did not differ significantly between the groups (3.4% vs. 1.5%, *p* = 0.370). During follow-up, 26 patients (7.2%) died and 39 (10.8%) experienced CCAEs. The causes of death were malignancy (*n* = 8), cerebrovascular disease (*n* = 3), pneumonia (*n* = 4), sepsis (*n* = 6), and cardiac causes (*n* = 5).

**Figure 2:**
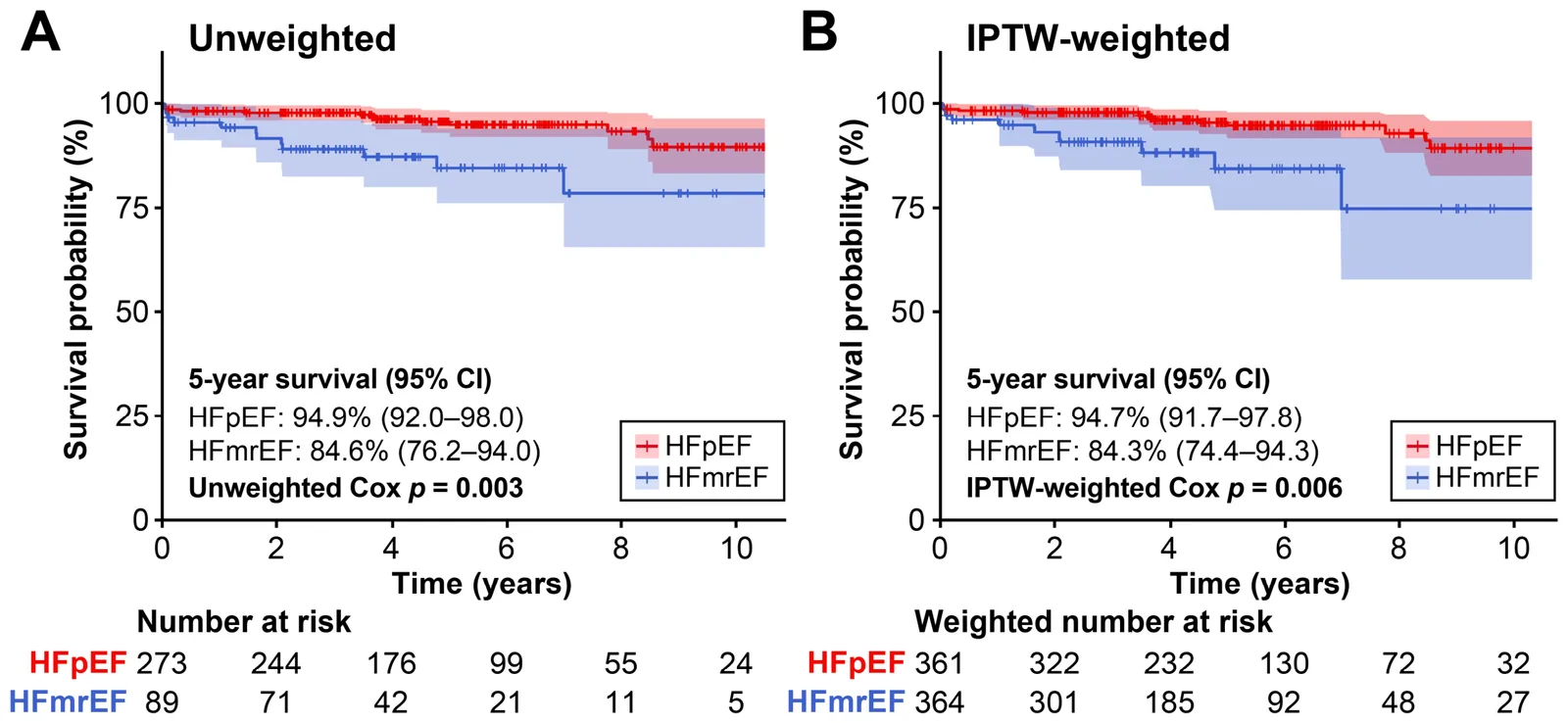
Overall survival according to HF phenotype before and after inverse probability of treatment weighting (IPTW): (**A**) unweighted Kaplan–Meier survival curves and (**B**) IPTW-weighted survival curves. Shaded areas represent 95% confidence intervals. *p* values were derived from an unweighted Cox proportional hazards model for panel (**A**) and an IPTW-weighted Cox proportional hazards model for panel (**B**). For panel (**B**), the values shown in the risk table are weighted risk-set totals (sums of IPTW weights among patients remaining at risk) rather than actual patient counts. HFpEF, heart failure with preserved ejection fraction; HFmrEF, heart failure with mildly reduced ejection fraction.

Overall survival was lower in the HFmrEF group than in the HFpEF group in both the unweighted and IPTW-weighted analyses (Figure 2). The 5-year survival rates were 84.6% (95% CI, 76.2–94.0) versus 94.9% (95% CI, 92.0–98.0) in the unweighted analysis and 84.3% (95% CI, 74.4–94.3) versus 94.7% (95% CI, 91.7–97.8) after IPTW, respectively. In the unweighted multivariable Cox model adjusted for age, chronic renal failure, and peripheral arterial disease, HFmrEF was associated with a higher risk of all-cause mortality (HR, 2.97; 95% CI, 1.36–6.46; *p* = 0.006). This association remained consistent in the IPTW-weighted Cox analysis (HR, 3.09; 95% CI, 1.39–6.89; *p* = 0.006) (Table 4).

**Figure 3:**
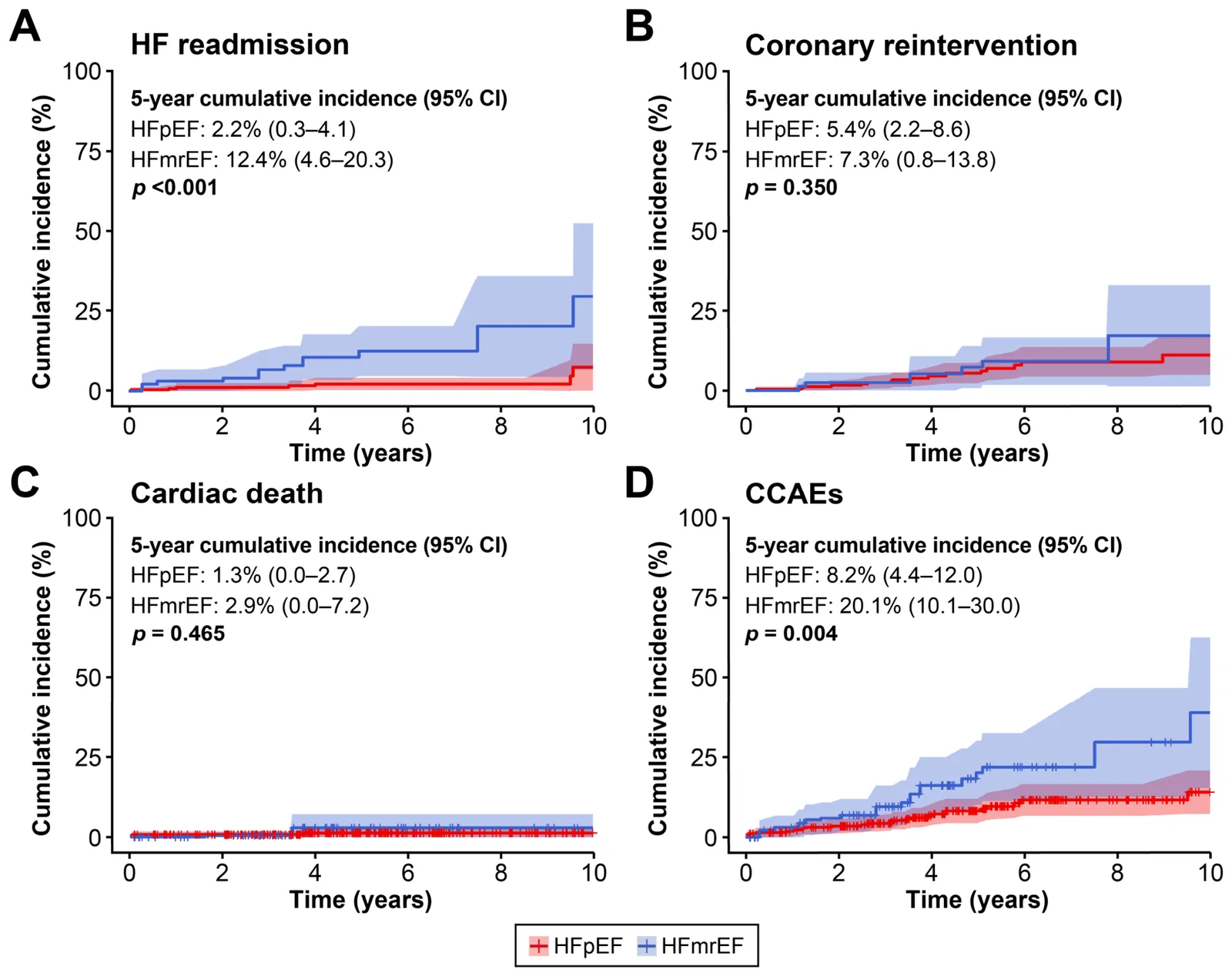
IPTW-weighted cumulative incidence functions for clinical outcomes in patients with HFpEF and HFmrEF: (**A**) HF readmission, (**B**) coronary reintervention, (**C**) cardiac death, and (**D**) composite cardiovascular adverse events (CCAEs). All-cause death was treated as the competing event for HF readmission and coronary reintervention, whereas non-cardiac death was treated as the competing event for cardiac death and CCAEs. Shaded areas represent 95% confidence intervals. *p* values were derived from IPTW-weighted Fine–Gray subdistribution hazard models. HFpEF, heart failure with preserved ejection fraction; HFmrEF, heart failure with mildly reduced ejection fraction; IPTW, inverse probability of treatment weighting.

In the IPTW-weighted competing-risk analyses (Figure 3), HF readmission occurred in 17 patients (4.7%). The 5-year cumulative incidence was 12.4% in the HFmrEF group and 2.2% in the HFpEF group. In the Fine–Gray analysis, HFmrEF was associated with a higher incidence of HF readmission (sHR, 5.72; 95% CI, 2.12–15.45; *p* < 0.001) (Figure 3A). In contrast, no statistically significant between-group differences were detected in coronary reintervention (sHR, 1.58; 95% CI, 0.60–4.14; *p* = 0.350) or cardiac death (sHR, 2.10; 95% CI, 0.29–15.38; *p* = 0.465) (Figure 3B,C).

The 5-year IPTW-weighted cumulative incidence of CCAEs was higher in the HFmrEF group than in the HFpEF group (20.1% vs. 8.2%). In the unweighted multivariable Fine–Gray model, HFmrEF was associated with a higher subdistribution hazard of CCAEs (sHR, 2.51; 95% CI, 1.32–4.78; *p* = 0.005), with a consistent association in the IPTW-weighted analysis (sHR, 2.67; 95% CI, 1.37–5.19; *p* = 0.004) (Figure 3D and Table 4).

### 3.3. Longitudinal Echocardiographic Changes

**Figure 4:**
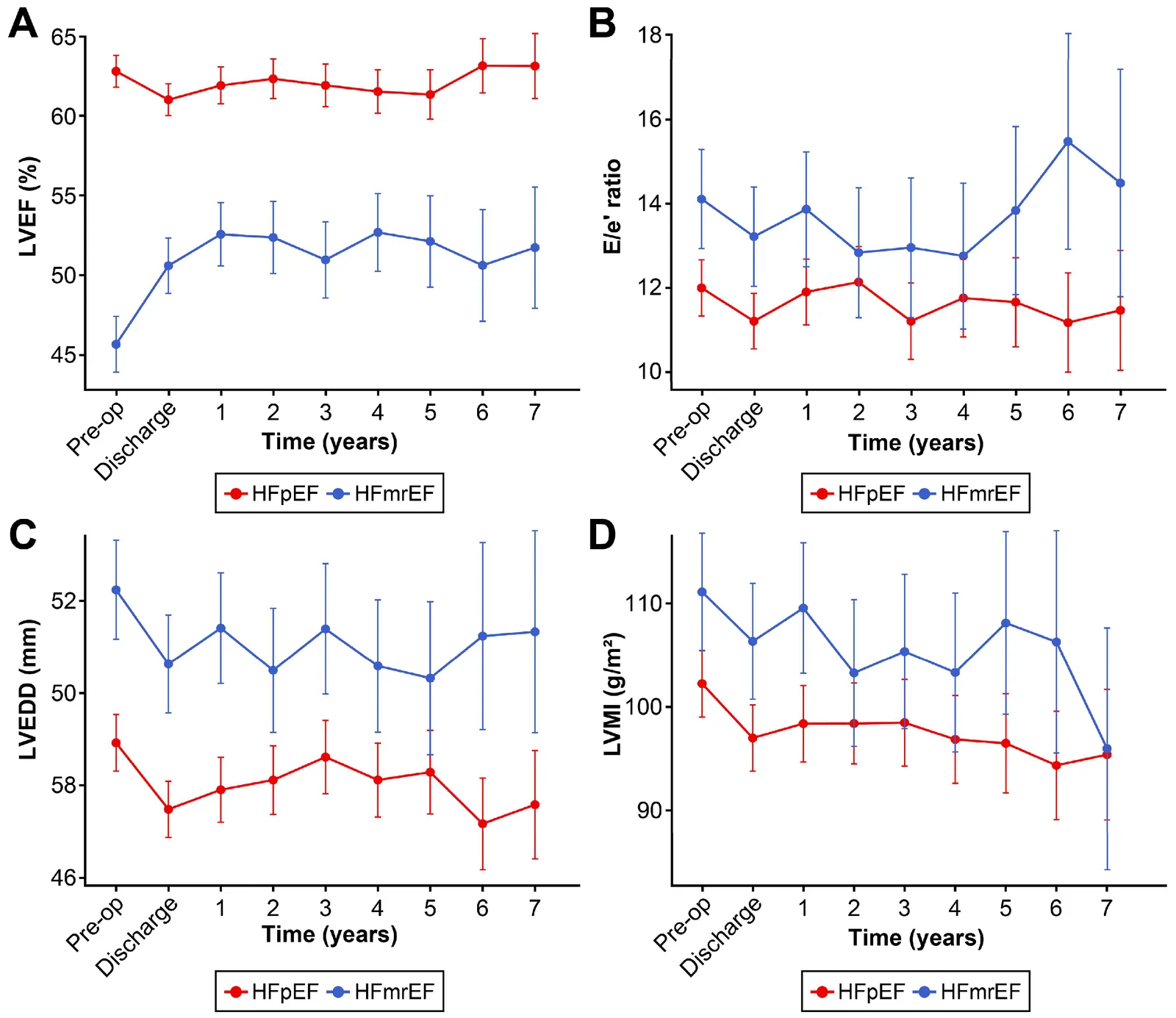
Echocardiographic trajectories in patients with HFpEF and HFmrEF. Estimated marginal means with 95% confidence intervals are presented for (**A**) left ventricular ejection fraction (LVEF), (**B**) E/e′, (**C**) left ventricular end-diastolic diameter (LVEDD), and (**D**) left ventricular mass index (LVMI). The graphical presentation was limited to 7 years because of the small number of observations at later time points. HFpEF, heart failure with preserved ejection fraction; HFmrEF, heart failure with mildly reduced ejection fraction.

In the linear mixed-effects models, significant group-by-time interactions were observed for LVEF and LVESD (both *p* < 0.001), indicating different longitudinal trajectories between the HFpEF and HFmrEF groups (Figure 4). In the HFmrEF group, the estimated marginal mean LVEF increased from 45.7% (95% CI, 43.9–47.4) preoperatively to 50.6% (95% CI, 48.8–52.3) at discharge. In contrast, no significant group-by-time interactions were observed for LVEDD (*p* = 0.205), LVMI (*p* = 0.534), or E/e′ (*p* = 0.486). Longitudinal echocardiographic trajectories through 7 years are shown in Figure 4, and the number of patients with available measurements at each time point through 9 years is provided in Supplementary Table S1.

## 4. Discussion

In this retrospective observational study, patients with HFmrEF who underwent OPCAB had lower overall survival and a higher incidence of CCAEs than those with HFpEF. HF readmission was more frequent in the HFmrEF group, whereas no statistically significant between-group difference in coronary reintervention was detected. LVEF showed a significant group-by-time interaction, with an early postoperative increase in the HFmrEF group, whereas no significant group-by-time interaction was observed for E/e′. Patients with HFmrEF also had greater baseline HF severity, characterized by higher NT-proBNP levels among those with available measurements and more adverse echocardiographic features. These differences suggest that HFmrEF may identify a higher-risk clinical phenotype rather than reflect the prognostic effect of EF category alone.

Among patients hospitalized with HF, approximately one-half have HFpEF or HFmrEF, and HFmrEF accounts for about 13–24% of HF cases [6,8,14,15]. HFmrEF, recognized relatively recently as a distinct HF phenotype, appears to occupy an intermediate position between HFrEF and HFpEF. Patients with HFmrEF may exhibit dynamic changes in LV function, with LVEF improving or declining over time in response to treatment and disease progression [8,16,17]. Given the substantial prevalence of CAD in patients with HFmrEF and HFpEF, there is growing interest in their clinical outcomes following coronary revascularization. In previous studies of patients undergoing CABG, HF or LV systolic dysfunction has often been classified using broader EF categories, potentially grouping patients with HFmrEF together with either HFrEF or HFpEF [9,10,18]. By specifically comparing patients with HFmrEF and HFpEF undergoing isolated OPCAB, the present study provides outcome data according to contemporary HF phenotypes in a population in which direct comparisons have been limited.

Our findings are broadly consistent with those of Deo et al. [19], who reported progressively poorer long-term outcomes after CABG across the HF spectrum with decreasing LVEF. In their study, patients classified as HFmrEF (LVEF, 40–55%) had a higher burden of recurrent HF hospitalization than those classified as HFpEF (LVEF, >55%). Similarly, patients with HFmrEF in our study had a higher cumulative incidence of HF readmission than those with HFpEF. Together, these findings suggest that patients with mildly reduced LVEF may remain vulnerable to recurrent HF events after surgical revascularization and warrant continued clinical follow-up after CABG.

Although Dalen et al. did not explicitly differentiate HFmrEF, their study demonstrated that both HF status and LVEF were associated with mortality after CABG, with reduced EF (<50%) showing a stronger association with early mortality than HF status [10]. Similarly, Sun et al. [18] reported that HF had important prognostic implications beyond LVEF in patients undergoing CABG. Together, these findings highlight the prognostic relevance of both HF status and LVEF in patients undergoing CABG. Given that only 5 of the 26 deaths in our study were cardiac and most were attributable to non-cardiac causes, the association between HFmrEF and all-cause mortality may reflect greater overall disease burden rather than an increased risk of cardiovascular mortality.

Thuijs et al. [11] used smoothing spline analysis and demonstrated a progressively increasing risk of mortality with decreasing LVEF below 50% after left main coronary revascularization (PCI or CABG), which is broadly consistent with our findings. However, LVEF is dynamic and may change over time in response to medical therapy, device therapy, and coronary revascularization. In our study, patients with HFmrEF showed an early postoperative increase in LVEF, with a longitudinal trajectory that differed significantly from that of patients with HFpEF. At discharge, among patients with preoperative HFmrEF, LVEF was ≥50% in 55.1%, 41–49% in 33.7%, and ≤40% in 11.2%. Previous studies have suggested that prognosis may differ according to longitudinal changes in LVEF, with improvement in EF generally associated with more favorable outcomes and deterioration with poorer outcomes [16,17,20]. However, because the timing and availability of follow-up echocardiography varied substantially among patients, whether this early increase was consistently maintained during long-term follow-up could not be reliably determined. We therefore could not reliably evaluate clinical outcomes according to subsequent EF trajectory. In addition, our 2012–2020 cohort largely predates the widespread adoption of contemporary guideline-directed therapy for HFmrEF and HFpEF, particularly SGLT2 inhibitors; therefore, our findings may not fully reflect outcomes under contemporary HF management [7,21]. Future prospective studies with standardized echocardiographic follow-up and contemporary HF-directed therapy are needed to clarify the prognostic significance of longitudinal changes in HF phenotype after CABG.

With respect to diastolic function, no significant group-by-time interaction was observed for E/e′, with limited change during follow-up. However, because E/e′ provides only an indirect estimate of LV filling pressure, this finding alone cannot establish persistent abnormalities in diastolic function after revascularization. Because HF has a multifactorial pathophysiology beyond obstructive CAD, mechanisms other than epicardial coronary disease may contribute to ongoing HF manifestations [5]. Coronary microvascular dysfunction and related microvascular abnormalities have been proposed as potential mechanisms contributing to HFpEF pathophysiology [22,23]. Although not directly assessed in our study, these mechanisms may represent hypothesis-generating explanations for persistent HF manifestations after revascularization. Future prospective studies incorporating comprehensive assessment of diastolic and coronary microvascular function may help clarify the mechanisms underlying HF manifestations after CABG.

This study has several limitations. First, the retrospective design may have introduced selection bias and incomplete clinical information. HF was retrospectively ascertained from medical records, and misclassification remains possible, particularly for HFpEF. Second, patients with HFmrEF had greater baseline HF severity, including higher NT-proBNP levels among those with available measurements and more adverse echocardiographic features. Although IPTW reduced measured confounding, residual confounding related to baseline HF severity and unmeasured factors remains possible. Third, the limited numbers of deaths (*n* = 26) and HF readmissions (*n* = 17) constrained multivariable adjustment and the precision of effect estimates; parsimonious models were therefore used to reduce the risk of overfitting. Moreover, only five deaths were cardiac, limiting inferences regarding cardiovascular mortality. Fourth, nonfatal outcomes were retrospectively ascertained from institutional medical records without independent adjudication, and events occurring exclusively at other institutions may have been missed. Detailed longitudinal data on HF-directed medical therapy were also not consistently available, precluding adequate adjustment for differences and temporal changes in medical therapy. Fifth, echocardiographic follow-up was nonuniform and became less available over time, limiting the assessment of longitudinal EF trajectories and their associations with clinical outcomes. The small number of patients remaining at risk during extended follow-up also reduced the precision of late outcome estimates. Finally, this single-center study included only patients undergoing isolated OPCAB, which may limit generalizability to patients undergoing on-pump CABG or to centers with different revascularization practices.

## 5. Conclusions

In patients with HF undergoing isolated OPCAB at a single center, HFmrEF was associated with lower overall survival and a higher incidence of CCAEs compared with HFpEF, with a higher incidence of HF readmission. Although an early postoperative increase in LVEF was observed in patients with HFmrEF, differences in baseline HF severity and potential residual confounding preclude attributing the observed differences in long-term outcomes to EF category alone. Further prospective studies are needed to clarify the prognostic significance of HF phenotype after CABG.

## Figures and Tables

**Table 1:** Baseline clinical characteristics and covariate balance before and after inverse probability of treatment weighting.

Variables ^a^	Before IPTW				After IPTW		
	HFpEF (*n* = 273)	HFmrEF (*n* = 89)	*p*-Value	Absolute SMD	HFpEF	HFmrEF	Absolute SMD
Age (years)	62.5 ± 10.6	63.2 ± 11.9	0.627	0.06	62.6 ± 10.5	62.3 ± 12.0	0.03
Female	65 (23.8%)	18 (20.2%)	0.580	0.09	23.1%	23.4%	0.01
Body mass index (kg/m^2^)	24.8 ± 3.2	24.1 ± 2.9	0.072	0.23	24.7 ± 3.2	24.8 ± 3.3	0.04
Smoking	125 (45.8%)	47 (52.8%)	0.303	0.14	47.5%	49.9%	0.05
Hypertension	195 (71.4%)	66 (74.2%)	0.717	0.06	72.1%	72.6%	0.01
Diabetes mellitus	121 (44.3%)	44 (49.4%)	0.472	0.10	45.6%	47.0%	0.03
Chronic renal failure	34 (12.5%)	14 (15.7%)	0.541	0.09	13.2%	12.5%	0.02
Cerebrovascular accident	35 (12.8%)	10 (11.2%)	0.835	0.05	12.9%	15.2%	0.07
Peripheral arterial disease	7 (2.6%)	8 (9.0%)	0.014	0.28	4.0%	4.0%	0.00
Dyslipidemia	47 (17.2%)	14 (15.7%)	0.871	0.04	16.8%	15.1%	0.05
Previous PCI	55 (20.1%)	11 (12.4%)	0.098	0.21	18.3%	17.5%	0.02
3-vessel disease	200 (73.3%)	70 (78.7%)	0.382	0.13	74.5%	74.9%	0.01
Left main disease	59 (21.6%)	19 (21.3%)	0.958	0.01	21.4%	21.2%	0.01

^a^ Values are presented as mean ± standard deviation or *n* (%), as appropriate. After IPTW, values represent weighted means or weighted proportions. Standardized mean differences (SMDs) were used to assess covariate balance, with an absolute SMD < 0.1 indicating adequate balance. HFmrEF, heart failure with mildly reduced ejection fraction; HFpEF, heart failure with preserved ejection fraction; IPTW, inverse probability of treatment weighting; PCI, percutaneous coronary intervention.

**Table 2:** Preoperative biomarker and echocardiographic characteristics.

Variables ^a^	All (*n* = 362)	HFpEF (*n* = 273)	HFmrEF (*n* = 89)	*p*-Value
**Biomarker**				
NT-proBNP (pg/mL)	328 (142, 1595)	178 (136, 619)	1909 (745, 8202)	<0.001
**Echocardiographic parameters**				
LVEF (%)	58.7 ± 10.2	62.8 ± 8.0	45.7 ± 2.4	<0.001
LVESD (mm)	33.2 ± 6.2	31.3 ± 4.9	39.3 ± 5.7	<0.001
LVEDD (mm)	49.7 ± 4.9	48.9 ± 4.3	52.2 ± 5.6	<0.001
LAVI (mL/m^2^)	35.6 ± 12.9	35.1 ± 11.1	37.9 ± 19.3	0.097
LVMI (g/m^2^)	104.4 ± 28.6	102.2 ± 28.1	111.1 ± 29.3	0.016
E/e′	12.3 ± 4.6	12.0 ± 4.6	14.1 ± 5.7	<0.001

^a^ Values are presented as mean ± standard deviation or median (interquartile range), as appropriate. NT-proBNP measurements were available in 234 patients (HFpEF, *n* = 171; HFmrEF, *n* = 63). Available preoperative echocardiographic measurements in the HFpEF and HFmrEF groups, respectively, were: LVEF, 273 and 89; LVESD, 264 and 86; LVEDD, 264 and 86; LAVI, 197 and 67; LVMI, 261 and 85; and E/e′, 247 and 80. HFmrEF, heart failure with mildly reduced ejection fraction; HFpEF, heart failure with preserved ejection fraction; LAVI, left atrial volume index; LVEDD, left ventricular end-diastolic diameter; LVEF, left ventricular ejection fraction; LVESD, left ventricular end-systolic diameter; LVMI, left ventricular mass index; NT-proBNP, N-terminal pro–B-type natriuretic peptide.

**Table 3:** Operative characteristics and clinical outcomes.

Variables ^a^	All (*n* = 362)	HFpEF (*n* = 273)	HFmrEF (*n* = 89)	*p*-Value
**Operative characteristics**				
Number of distal anastomoses	3.10 ± 1.00	3.11 ± 1.01	3.07 ± 1.00	0.775
Composite graft	49 (13.5)	39 (14.3)	10 (11.2)	0.465
Aortocoronary graft	332 (91.7)	249 (91.2)	83 (93.3)	0.542
Complete revascularization	334 (92.3)	256 (93.8)	78 (87.6)	0.069
Operative time (min)	296.0 ± 66.9	294.8 ± 65.3	299.8 ± 72.0	0.536
**Early clinical outcomes**				
Reoperation for bleeding	1 (0.3)	1 (0.4)	0 (0)	>0.999
Stroke	4 (1.1)	3 (1.1)	1 (1.1)	>0.999
Prolonged ventilation (>72 h)	18 (5.0)	10 (3.7)	8 (9.0)	0.053
Newly required dialysis	12 (3.3)	5 (1.8)	7 (7.9)	0.012
Postoperative atrial fibrillation	34 (9.4)	25 (9.2)	9 (10.1)	0.789
In-hospital mortality	7 (1.9)	4 (1.5)	3 (3.4)	0.370
**Long-term clinical outcomes**				
All-cause death	26 (7.2)	14 (5.1)	12 (13.5)	-
Non-cardiac death	21 (5.8)	11 (4.0)	10 (11.2)	-
CCAEs	39 (10.8)	23 (8.4)	16 (18.0)	-
Cardiac death	5 (1.4)	3 (1.1)	2 (2.2)	-
HF readmission	17 (4.7)	7 (2.6)	10 (11.2)	-
Coronary reintervention	22 (6.1)	16 (5.9)	6 (6.7)	-

^a^ Values are presented as mean ± standard deviation or *n* (%). All values are based on the original, unweighted study population. Individual patients could experience more than one CCAE component during follow-up; therefore, the sum of individual components may exceed the number of patients experiencing a CCAE. CCAEs, composite cardiovascular adverse events; HFmrEF, heart failure with mildly reduced ejection fraction; HFpEF, heart failure with preserved ejection fraction.

**Table 4:** Cox and Fine–Gray models of clinical outcomes before and after inverse probability of treatment weighting.

Variables	Overall Survival		CCAEs	
	HR (95% CI)	*p*-Value	sHR (95% CI)	*p*-Value
**Unweighted analysis**				
HFmrEF vs. HFpEF	2.97 (1.36–6.46)	0.006	2.51 (1.32–4.78)	0.005
Age, years	1.03 (0.99–1.07)	0.091	1.03 (1.00–1.06)	0.068
Chronic renal failure	2.13 (0.87–5.23)	0.099	2.44 (1.08–5.49)	0.031
Peripheral arterial disease	2.71 (0.82–8.99)	0.103	1.68 (0.40–7.02)	0.478
**IPTW-weighted analysis**				
HFmrEF vs. HFpEF	3.09 (1.39–6.89)	0.006	2.67 (1.37–5.19)	0.004

HRs for overall survival were estimated using Cox models and sHRs for CCAEs using Fine–Gray models. Unweighted models were adjusted for age, chronic renal failure, and peripheral arterial disease. After IPTW, HF phenotype was evaluated without additional covariate adjustment. HFpEF was the reference group, and non-cardiac death was the competing event for CCAEs. CCAEs, composite cardiovascular adverse events; CI, confidence interval; HFmrEF, heart failure with mildly reduced ejection fraction; HFpEF, heart failure with preserved ejection fraction; HR, hazard ratio; IPTW, inverse probability of treatment weighting; sHR, subdistribution hazard ratio.

## Data Availability

The data presented in this study are available on request from the corresponding author. The data are not publicly available due to privacy.

## Supplementary Materials

The following supporting information can be downloaded at: https://www.mdpi.com/article/10.3390/jcm15186946/s1, Table S1: Longitudinal changes in echocardiographic parameters; Figure S1: Distribution of propensity scores according to HF phenotype; Figure S2: Covariate balance before and after inverse probability of treatment weighting.

## Abbreviations

The following abbreviations are used in this manuscript:

HF	heart failure
CABG	coronary artery bypass grafting
CAD	coronary artery disease
LV	left ventricular
EF	ejection fraction
HFmrEF	HF with mildly reduced EF
HFpEF	HF with preserved EF
HFrEF	HF with reduced EF
CCAEs	composite cardiovascular adverse events
OPCAB	off-pump CABG
ITAs	internal thoracic arteries
PCI	percutaneous coronary intervention
