# Supplementary material for: Comparative Outcomes of Heart Failure with Preserved vs. Mildly Reduced Ejection Fraction After Off-Pump Coronary Artery Bypass: A Single-Center Retrospective Study
Source: J Clin Med. 2026 Sep 8;15(18):6946. doi: 10.3390/jcm15186946 (PMC13607658; doi:10.3390/jcm15186946)
Supplement: Supplementary file 1 [file jcm-15-06946-s001.zip › jcm-4480681-supplementary.pdf]

Table S1. Longitudinal changes in echocardiographic parameters

	Preoperative	Discharge	1y	2y	3y	4y	5y	6y	7y	8y	9y	*p* for Group ×Time	*p* for Group	*p* for Time
**LVEF (%)**												<0.001	<0.001	0.002
HFpEF	62.8 (61.8–63.8) [273]	61.0 (60.0–62.0) [271]	61.9 (60.7–63.0) [158]	62.3 (61.1–63.5) [133]	61.9 (60.6–63.2) [109]	61.5 (60.1–62.9) [107]	61.3 (59.8–62.9) [75]	63.1 (61.4–64.8) [60]	63.1 (61.1–65.1) [39]	60.9 (58.7–63.2) [33]	61.1 (58.7–63.5) [28]			
HFmrEF	45.7 (43.9–47.4) [89]	50.6 (48.8–52.3) [89]	52.5 (50.6–54.5) [56]	52.3 (50.1–54.6) [39]	50.9 (48.6–53.3) [34]	52.7 (50.2–55.1) [33]	52.1 (49.2–55.0) [22]	50.6 (47.1–54.1) [13]	51.7 (47.9–55.5) [11]	48.4 (43.7–53.1) [7]	48.0 (43.5–52.5) [8]			
**LVESD (mm)**												<0.001	<0.001	0.496
HFpEF	31.3 (30.6–32.0) [264]	31.5 (30.9–32.2) [271]	31.0 (30.2–31.7) [158]	31.1 (30.3–32.0) [133]	31.6 (30.7–32.5) [109]	31.6 (30.7–32.5) [107]	31.9 (30.8–32.9) [75]	30.6 (29.5–31.7) [60]	30.9 (29.6–32.2) [39]	31.7 (30.3–33.1) [33]	31.3 (29.7–32.8) [28]			
HFmrEF	39.3 (38.1–40.5) [86]	37.4 (36.2–38.6) [89]	37.1 (35.7–38.4) [56]	36.5 (34.9–38.0) [39]	37.4 (35.8–39.0) [34]	36.0 (34.4–37.6) [33]	36.5 (34.6–38.4) [22]	37.6 (35.4–39.9) [13]	35.9 (33.4–38.4) [11]	40.3 (37.3–43.4) [7]	38.9 (36.0–41.8) [8]			
**LVEDD (mm)**												0.205	<0.001	<0.001
HFpEF	48.9 (48.3–49.5) [264]	47.5 (46.9–48.1) [271]	47.9 (47.2–48.6) [158]	48.1 (47.4–48.8) [133]	48.6 (47.8–49.4) [109]	48.1 (47.3–48.9) [107]	48.3 (47.4–49.2) [75]	47.2 (46.2–48.1) [60]	47.6 (46.4–48.7) [39]	48.6 (47.4–49.9) [33]	47.1 (45.8–48.5) [28]			
HFmrEF	52.2 (51.2–53.3) [86]	50.6 (49.6–51.7) [89]	51.4 (50.2–52.6) [56]	50.5 (49.1–51.8) [39]	51.4 (50.0–52.8) [34]	50.6 (49.1–52.0) [33]	50.3 (48.7–52.0) [22]	51.2 (49.2–53.3) [13]	51.3 (49.1–53.5) [11]	51.7 (49.0–54.3) [7]	53.9 (51.3–56.4) [8]			
**LVMI (g/m²)**												0.534	0.008	0.004
HFpEF	102.2 (99.0–105.5) [261]	97.0 (93.8–100.2) [270]	98.4 (94.7–102.1) [157]	98.4 (94.5–102.3) [133]	98.5 (94.3–102.7) [109]	96.9 (92.6–101.1) [107]	96.5 (91.7–101.3) [75]	94.3 (89.1–99.6) [60]	95.4 (89.1–101.7) [38]	92.9 (86.2–99.7) [33]	90.8 (83.5–98.1) [28]			
HFmrEF	111.1 (105.5–116.8) [85]	106.4 (100.8–111.9) [89]	109.6 (103.2–115.9) [56]	103.3 (96.2–110.4) [39]	105.4 (97.9–112.8) [34]	103.3 (95.6–111.0) [32]	108.1 (99.3–117.0) [22]	106.3 (95.5–117.0) [13]	96.0 (84.3–107.6) [11]	95.3 (81.0–109.5) [7]	108.9 (95.2–122.5) [8]			
**E/E'**												0.486	0.002	0.234
HFpEF	12.0 (11.3–12.7) [247]	11.2 (10.6–11.9) [258]	11.9 (11.1–12.7) [155]	12.1 (11.3–13.0) [126]	11.2 (10.3–12.1) [104]	11.8 (10.8–12.7) [101]	11.7 (10.6–12.7) [72]	11.2 (10.0–12.3) [56]	11.5 (10.0–12.9) [36]	12.2 (10.7–13.7) [33]	12.0 (10.4–13.7) [27]			
HFmrEF	14.1 (12.9–15.3) [80]	13.2 (12.0–14.4) [78]	13.9 (12.5–15.2) [51]	12.8 (11.3–14.4) [37]	12.9 (11.3–14.6) [31]	12.8 (11.0–14.5) [28]	13.8 (11.8–15.8) [20]	15.5 (12.9–18.0) [11]	14.5 (11.8–17.2) [10]	13.1 (9.7–16.5) [6]	12.0 (8.6–15.4) [6]			

Values are presented as estimated marginal means (95% confidence intervals) derived from linear mixed-effects models. Numbers in brackets indicate the number of patients with available measurements at each time point. The models included group, time, and the group-by-time interaction as fixed effects and patient as a random effect, with time treated as a categorical variable. HFmrEF, heart failure with mildly reduced ejection fraction; HFpEF, heart failure with preserved ejection fraction; LVEDD, left ventricular end-diastolic diameter; LVEF, left ventricular ejection fraction; LVESD, left ventricular end-systolic diameter; LVMI, left ventricular mass index.

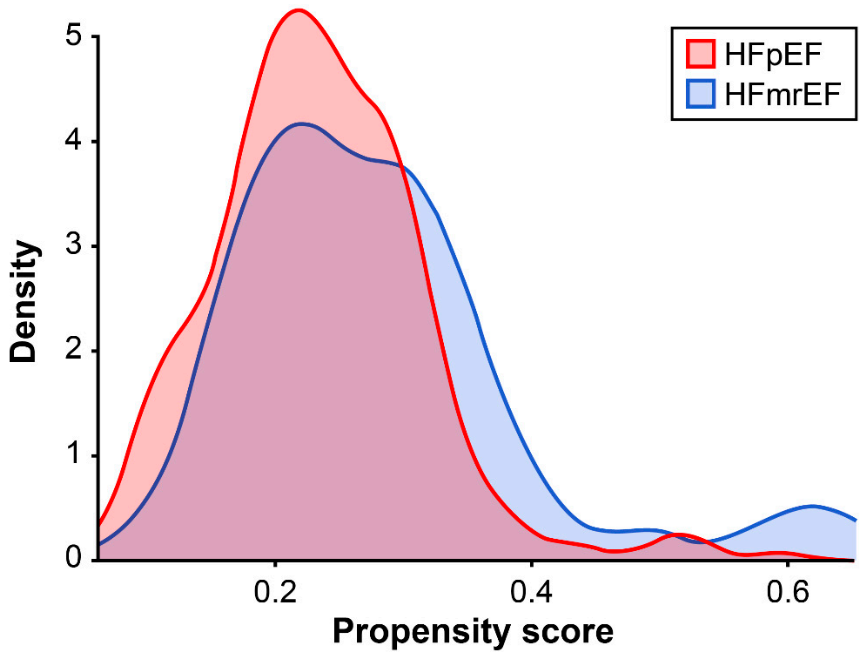

Figure S1. Distribution of propensity scores according to HF phenotype.

Density distributions of estimated propensity scores are shown for the HFpEF and HFmrEF groups, demonstrating overlap between the two groups. HFpEF, heart failure with preserved ejection fraction; HFmrEF, heart failure with mildly reduced ejection fraction.

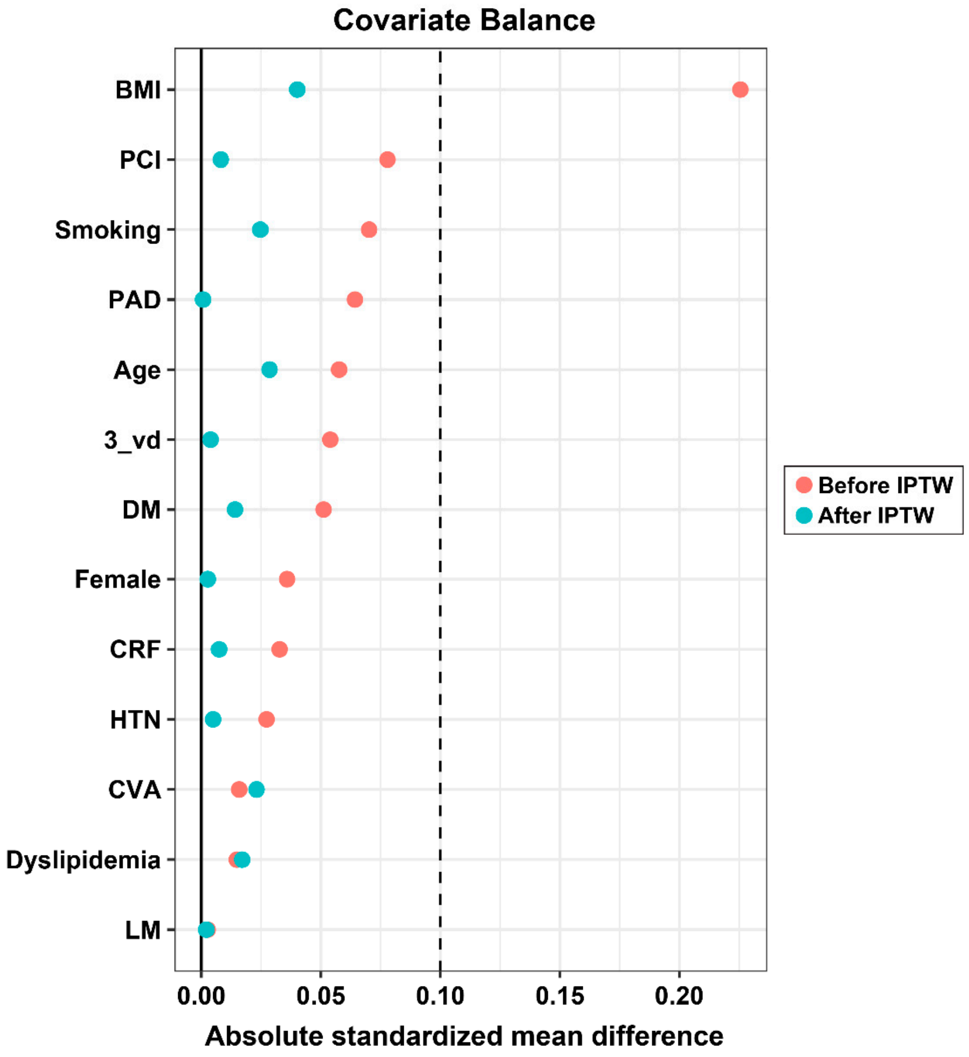

Figure S2. Covariate balance before and after inverse probability of treatment weighting.

Absolute standardized mean differences (SMDs) for baseline covariates between the HFpEF and HFmrEF groups are shown before and after IPTW. The dashed vertical line indicates an absolute SMD of 0.1, which was used as the threshold for adequate covariate balance. After IPTW, all included covariates had absolute SMDs <0.1. BMI, body mass index; CRF, chronic renal failure; CVA, cerebrovascular accident; DM, diabetes mellitus; HFmrEF, heart failure with mildly reduced ejection fraction; HFpEF, heart failure with preserved ejection fraction; HTN, hypertension; IPTW, inverse probability of treatment weighting; LM, left main disease; PAD, peripheral arterial disease; PCI, percutaneous coronary intervention; SMD, standardized mean difference; 3_vd; 3-vessel disease.
